# Notes of Experiments on the Effects of Woorara Introduced into the Stomach

**Published:** 1859-07

**Authors:** Daniel Brainard

**Affiliations:** Professor of Surgery in the College at Chicago, Illinois


*Notes of Experiments on the Effects of Woorara Introduced into the Stomach.* By DANIEL BRAINARD, M. D., Professor of Surgery in the College at Chicago, Illinois.

It is generally believed, that certain poisons, readily absorbed, and quickly fatal when applied to recent wounds, are entirely innocuous when taken into the stomach. The venom of serpents is referred to as the type of this class; and the South American poison, *curara*, *woorara*, etc., is in this respect ranked with it.

This belief, universal, it is said, in South America, seems to be founded on the fact, that bites of serpents and wounds from poisoned arrows, have been treated by sucking, without injury; and that game killed by poisoned weapons is eaten without ill effects; but in recent times it has received a confirmation, from the views of Messrs. Bernard and Pelouze, sanctioned by the Academy of Sciences, which has caused it to be regarded as one of the established facts of science; certain physiological doctrines and theories of the action of medicinal substances even have been built upon it. Hence its importance.

It will be perceived, on a moment's reflection, that this supposed fact is contrary to all analogies in physiology.

That any substance whatever should be absorbed uniformly and quickly from wounds, and cause death, and be in solution entirely incapable of being taken up by the gastro-intestinal mucous membrane, implies in this latter tissue a power of choosing and of rejecting, which modern researches have demonstrated it not to possess.

Facts also exist, unnoticed for the most part, militating strongly against this belief. Fontana found that the venom of the viper, when applied to the eyes of pigeons, caused the lids to swell so as to close them.* This led him to doubt its entire want of action when applied to mucous surfaces. The following is his description of his experiment to determine this question:

"Chance having presented me a great number of vipers, large and vigorous, I was unwilling to let the opportunity pass of deciding, for the benefit of posterity, so important a point of natural history." "I cut the heads off eight vipers, and expressed their venom, which was received in a small spoon, of which there may have been thirty drops and more. I introduced the whole of it within the beak and esophagus of a pigeon, which had fasted for eight hours. In less than a minute it appeared much weakened; two minutes after it began to vacillate; fell at length upon its side, with a sort of convulsions, and in six minutes was dead. The beak, the esophagus, and the crop, even to the gullet, were inflamed and livid, and the blood appeared blacker than ordinary. These parts were so much dis-

---

* *Traite du Venin de la Vipere*, etc. Florence, 1781. Vol. 2, page 307, Supplement.

colored, that they appeared to approach a state of mortification and gangrene."*

It would, no doubt, have surprised the laborious and accurate Fontana, whose discoveries many recent observers have often reproduced, without in the least being aware of it, if he could have foreseen that his authority would be invoked, as it is in some recent works of great general accuracy, in support of the other side of this question, which he flattered himself to be settling for posterity.†

With the South-American poison of the Amazon, the *Ticunas*, the experiments of Fontana were far more numerous and conclusive.

"I made a pigeon swallow six grains of this poison, and it died in twenty-five minutes. I repeated this experiment on two other pigeons, and they both died in thirty minutes."‡

These experiments were repeated on six rabbits, three Guinea pigs, and two pigeons, with the same results. Fontana adds:

"I have observed in general, in making the above experiments, that the animals died with more difficulty, or were unaffected, if the stomach was full when the poison was swallowed;" and concluded as follows: "I deduce as an established fact (*verite de fait*), that the American poison, taken internally, is a poison; but that a sensible quantity of it is required to kill even a small animal."

Four, and even six grains, were found by Fontana to have little or no effect when swallowed by rabbits.

M. Vulpian found a concentrated solution of curara, applied upon the back of a frog, caused death in four or five hours.§

In 1854, while ignorant of the above views, I formed and published the opinion that curara is absorbed from the stomach of living animals. I introduced five grains of it, dissolved in two drachms of distilled water, into the stomach of a pigeon, which had been fasting for twenty-four hours. The bird remained twenty-four hours longer without food or drink. I then killed it; no fluid was found in the intestinal canal. The whole length of this was carefully washed with one drachm of distilled water, and this injected under the skin of another pigeon. No effect was produced. This experiment was repeated on two other birds, with the same results. As the quantity employed was sufficient to kill twenty-five pigeons, in five minutes, when injected under the skin, it is difficult to avoid the conclusion, that it is absorbed.‖

These experiments, while they demonstrated to my satisfaction the fact of absorption, misled me entirely as to the perfect inactivity of the poison swallowed in large quantity.

1858. I injected six grains of curara, dissolved in two drachms of water, into the stomach of a rat. In twenty-five minutes he began to be affected, and died in forty-five minutes. This experiment was repeated

---

* *Vide op. cit.* † *Ib.* page 90.

‡ See Berard's Physiology, vol. 2, Art. Absorption.

§ *Gazette Médicale* for 1858, p. 638.

‖ Essay on a New Method of Treating Serpent Bites and other Poisoned Wounds. Chicago, 1854; p. 17.

upon two other rats, with the same effect. In both the stomach was found distended with air, and the right side of the heart with black blood.

In these experiments, I took especial pains to prevent any of the solution from entering the larynx, as it is well known that the bronchial mucous surface absorbs much more rapidly than does the gastro-intestinal; and I withhold several experiments on rats and Guinea pigs, because I could not be sure that a minute portion of the solution had not entered the air passages. While feeling sure, then, that the solution of woorara is absorbed from the gastro-intestinal mucous membrane of birds and rats, with sufficient rapidity to cause death quickly, I have not found the same to be the case with rabbits, in which I have injected as much as thirty grains in solution, without giving rise to severe effects.

This insusceptibility of the stomach of certain classes of animals to the action of poisons, is a fact well known, and depends upon the absorbing power of the tissue. The absorbing power depends upon the thickness, vascularity, and density of the part, and the rapidity of the circulation. The bronchial mucous membrane is fine, and very vascular, and therefore absorbs all poisons, including woorara, rapidly. The coats of the blood-vessels also absorb quickly. Both these tissu·s absorb much more rapidly than the stomach, which, being covered with mucus, takes up most solutions more slowly.

The stomach of the *carnivora* absorbs more rapidly than the first stomach of the ruminants, or the crop of birds; so that it often happens that these animals take noxious substances with the food, with impunity. An example of this, often cited, is that of the goat eating the cicuta, without injury.

The skin absorbs more slowly than the mucous membrane of the stomach—being covered with dry epithelium. The quality of the solution also has an effect upon the capacity of absorption.

All that can be inferred from the experiments of Bernard, is, that in dead animal membranes absorption is very slow. Experiments on dead membranes authorize no conclusion in regard to the living; the activity of the imbibing power being much greater in these latter.

This doctrine, that there are certain substances in solution, which exert no great cauterizing or chemical action on the stomach, which are not absorbed in the least degree by it, but which are readily taken up by other membranes, is a retrograde step in physiology, and ought to be scrutinized before being adopted. Orfila, in the last edition of his Treatise on Toxicology, seems to adopt it. Taylor, who, in the first edition of his work on poisons (1848), stated that woorara, in a large dose, is a poison for all animals, has, in the recent edition of the same work, given the views of Bernard, without comment.

Carpenter, and other physiological writers, also adopt it. Under these circumstances, there seems little use in combating an error so deeply rooted; but this consideration should not prevent us from adhering to and promulgating what we deem the true doctrine of absorption.

Further experiments are required to determine many facts in regard to absorption. Does the membrane, by which a substance may be absorbed, exert an influence over its action afterwards, on the fluids or solids of the economy?

The nature of the active principle of woorara cannot be considered as entirely determined. Boussingault, in 1827, made an analysis of it, in which he detected an uncrystalizable substance, called curarina. Subsequent examinations seem to have confirmed the result of his analysis. Pereira, and nearly all the English authorities, state that *strychnos toxifera* yields the base of it; but Taylor, in the recent edition of his work, asserts that the plant from which it is prepared, is the *cocculus Amazonum*, and does not belong to the genus strychnos.

In my "Essay on a New Method of Treating Serpent Bite," &c., expressed the opinion, that the woorara acts in the same manner as the serpent-venom, and is probably identical with it. More numerous and careful observations, while they have confirmed my views of the similarity of the mode of action of these two poisons, have also disclosed differences which seem to forbid the positive conclusion that they are, *in every respect*, identical. Without dwelling upon these differences at length, it may be sufficient to state, that the woorara never produces the dark discoloration in wounds, which is so constant and characteristic an effect of serpent-bite.

The facts and experiments herein adduced seem to justify the following conclusion:

1. Woorara (curara), contrary to the received opinion, is absorbed from the stomach of birds, &c., often, under favorable circumstances, with sufficient rapidity to cause death.

